# A Bout of High Intensity Interval Training Lengthened Nerve Conduction Latency to the Non-exercised Affected Limb in Chronic Stroke

**DOI:** 10.3389/fphys.2018.00827

**Published:** 2018-07-02

**Authors:** Beraki Abraha, Arthur R. Chaves, Liam P. Kelly, Elizabeth M. Wallack, Katie P. Wadden, Jason McCarthy, Michelle Ploughman

**Affiliations:** Recovery and Performance Lab, Faculty of Medicine, Memorial University of Newfoundland, St. John's, NL, Canada

**Keywords:** transcranial magnetic stimulation, corticospinal excitability, neuroplasticity, aerobic exercise, stroke, rehabilitation, upper limb, high intensity interval training

## Abstract

**Objective:** Evaluate intensity-dependent effects of a single bout of high intensity interval training (HIIT) compared to moderate intensity constant-load exercise (MICE) on corticospinal excitability (CSE) and effects on upper limb performance in chronic stroke.

**Design:** Randomized cross-over trial.

**Setting:** Research laboratory in a tertiary rehabilitation hospital.

**Participants:** Convenience sample of 12 chronic stroke survivors.

**Outcome measures:** Bilateral CSE measures of intracortical inhibition and facilitation, motor thresholds, and motor evoked potential (MEP) latency using transcranial magnetic stimulation. Upper limb functional measures of dexterity (Box and Blocks Test) and strength (pinch and grip strength).

**Results:** Twelve (10 males; 62.50 ± 9.0 years old) chronic stroke (26.70 ± 23.0 months) survivors with moderate level of residual impairment participated. MEP latency from the ipsilesional hemisphere was lengthened after HIIT (pre: 24.27 ± 1.8 ms, and post: 25.04 ± 1.8 ms, *p* = 0.01) but not MICE (pre: 25.49 ± 1.10 ms, and post: 25.28 ± 1.0 ms, *p* = 0.44). There were no significant changes in motor thresholds, intracortical inhibition or facilitation. Pinch strength of the affected hand decreased after MICE (pre: 8.96 ± 1.9 kg vs. post: 8.40 ± 2.0 kg, *p* = 0.02) but not after HIIT (pre: 8.83 ± 2.0 kg vs. post: 8.65 ± 2.2 kg, *p* = 0.29). Regardless of type of aerobic exercise, higher total energy expenditure was associated with greater increases in pinch strength in the affected hand after exercise (*R*^2^ = 0.31, *p* = 0.04) and decreases in pinch strength of the less affected hand (*R*^2^ = 0.26 *p* = 0.02).

**Conclusion:** A single bout of HIIT resulted in lengthened nerve conduction latency in the affected hand that was not engaged in the exercise. Longer latency could be related to the cross-over effects of fatiguing exercise or to reduced hand spasticity. Somewhat counterintuitively, pinch strength of the affected hand decreased after MICE but not HIIT. Regardless of the structure of exercise, higher energy expended was associated with pinch strength gains in the affected hand and strength losses in the less affected hand. Since aerobic exercise has acute effects on MEP latency and hand strength, it could be paired with upper limb training to potentiate beneficial effects.

## Introduction

Stroke is the primary cause of adult onset disability (Krueger et al., 2015). In the acute phase of stroke, spontaneous recovery occurs as neural connections in the lesion-disrupted brain are created within a time-limited window known as the sensitive period (Nudo, 2013; Dromerick et al., 2015; Zeiler et al., 2016; Ward, 2017). Later, in the chronic phase of stroke, the brain's ability to undergo neuroplasticity is diminished, and a plateau in recovery marks the closure of the sensitive period, further reducing an individual's capacity to recover from residual impairments (Dromerick et al., 2015; Ward, 2017). In the chronic phase of stroke, advanced stroke rehabilitation techniques to foster neuroplasticity mechanisms have been developed, such as constraint-induced therapy (Ploughman and Corbett, 2004), virtual reality (Saposnik et al., 2016), robotic assistive devices, and brain stimulation methods (Pollock et al., 2014; Hatem et al., 2016). However, to date these rehabilitation techniques produce small to moderate effects on motor recovery (Pollock et al., 2014; Hatem et al., 2016). Therefore, there is a need to develop better stroke rehabilitation interventions that promote neuroplasticity and help stroke survivors overcome the recovery plateau.

Aerobic exercise is a cost-effective intervention that upregulates markers of brain health (Cotman and Berchtold, 2002; Ploughman, 2008; Austin et al., 2014; Ploughman et al., 2015). Aerobic exercise is believed to mediate neurotrophins; growth-promoting factors that stimulate synaptogenesis, dendritic branching, and long-term potentiation (da Silva et al., 2016). These are critical processes for improving the acquisition of new skills, learning, and memory, and therefore have important implications in clinical populations such as stroke (Bliss and Cooke, 2011; Zeiler and Krakauer, 2013). Presently, there is a focus on determining the specifics of aerobic exercise prescription to optimally prime the brain (Ploughman et al., 2005, 2008; Singh et al., 2014; Robertson et al., 2015; Saucedo Marquez et al., 2015; Hwang et al., 2016; Charalambous et al., 2017; Gentil and Del Vecchio, 2017; Kelly et al., 2017; Morais et al., 2017; Nepveu et al., 2017; Neva et al., 2017). The benefits of aerobic exercise appear to be intensity dependent (Ploughman et al., 2007b; Hasan et al., 2016; Kelly et al., 2017) and several studies have shown that delivering high-intensity aerobic exercise protocols is superior to low or moderate intensity exercise (Ferris et al., 2007; Rojas et al., 2014; Saucedo Marquez et al., 2015; Hussain et al., 2016; MacInnis and Gibala, 2016). In addition to the delivery of aerobic exercise at specific intensities, the ratio of work-to-rest is an important consideration for clinical populations. *High-intensity interval training* (HIIT) is a type of exercise protocol that has been used for deconditioned and clinical populations who are unable to maintain continuous exercise (Lucas et al., 2015; Boyne et al., 2016; Hussain et al., 2016; Ito et al., 2016; Ribeiro et al., 2017). HIIT is an intermittent type of training that may be an advantageous aerobic exercise protocol for individuals with stroke because bouts of high-intensity exercise (work) are separated by varying amounts of passive (rest) or active (lower intensity) recovery periods (Gibala and Jones, 2013). Nonetheless, when considering the impact of aerobic exercise prescription on physiological and clinical measures, few studies have investigated the differential effect of HIIT compared to *moderate intensity continuous exercise* (MICE) in individuals with stroke.

Motor recovery and function in both acute and chronic stroke can be predicted by assessing the excitability of the corticospinal tract with transcranial magnetic stimulation (TMS) (Stinear et al., 2007; Di Pino et al., 2014). In healthy populations, evidence from TMS studies suggest that an acute bout of aerobic exercise modulates measures of corticospinal excitability (CSE) (Singh et al., 2014; Smith et al., 2014). In the field of stroke rehabilitation, there is accumulating evidence that aerobic exercise can be combined with other therapies to modulate measures of CSE and improve function (Ploughman et al., 2007a, 2008, 2009; Hendrikse et al., 2017). However, when evaluating measures of CSE, less is known about the optimal aerobic exercise prescription (i.e., frequency, intensity, type, and time) to promote neuroplasticity (Austin et al., 2014; Ploughman et al., 2015; Hasan et al., 2016). In chronic stroke, Nepveu et al. (2017), showed that a single bout of aerobic exercise reaching maximal exertion levels (i.e., maximal graded exercise test) was sufficient to change the ratio of cortical inhibition between the ipsilesional and contralesional hemispheres (Nepveu et al., 2017). The authors argued that the reduced cortical inhibition was the result of decreased C-aminobutyric acid (GABAergic) activity in the ipsilesional hemisphere following maximal aerobic exercise. These GABAergic neurons are primarily responsible for cortical inhibition and decreasing their activity is essential for long-term potentiation-like processes that lead to learning (Bachtiar and Stagg, 2014). Accordingly, motor skill acquisition and retention are also enhanced after a single bout of HIIT in individuals with stroke (Nepveu et al., 2017). Taken together, aerobic exercise seems to be a beneficial intervention for modulating brain mechanisms related to neuroplasticity in chronic stroke.

To evaluate intensity dependent effects on physiological outcomes using different delivery methods of aerobic exercise (i.e., HIIT vs. MICE), it is important to match the total amount of work performed. Energy expenditure of exercise must be controlled to ensure changes in CSE are due to the characteristics of exercise (i.e., intensity-level, work-to-rest ratio), or conversely, simply due to total work performed (Henderson et al., 2007). Previous research in multiple sclerosis showed that CSE differences noticed between MICE vs. HIIT may have occurred due to total work performed, rather than the type of exercise itself (Collett et al., 2017). Previous studies that investigated CSE changes in stroke after HIIT vs. MICE did not control for the total amount of work performed between interventions, and therefore, it is difficult to know what characteristics of exercise resulted in CSE changes (intensity or structure). The primary objective of the current study was to evaluate changes in CSE after single bouts of MICE and HIIT when matched for total energy expenditure of exercise. Additionally, it is currently unknown how acute exercise-induced changes in CSE relate to measures of hand function in stroke survivors. Therefore, the secondary aim was to investigate relationships between CSE changes and clinical measures of hand dexterity (Box and Blocks Test) and strength (pinch and grip). It was hypothesized that HIIT would have a greater effect on CSE and clinical measure of hand function than MICE.

## Materials and methods

### Ethics

The current study was approved by the Health Research Ethics Authority of Newfoundland and Labrador (file number 20162291) and was carried out in accordance with the Declaration of Helsinki on the use of human subjects in experiments. Subjects provided written informed consent before study participation.

### Study design

A crossover study design was used to evaluate intensity dependent changes in CSE, and upper limb performance after two different bouts of aerobic exercise that were matched for total energy expenditure. The aerobic exercise bouts consisted of MICE and HIIT, which were performed in randomized order with at least 7-days between sessions to avoid possible carry-over effects (Kuo et al., 2005; Kelly and Basset, 2017). Participants first completed a maximal graded exercise test to determine maximal oxygen uptake (VO_2max_), which was used to assign workloads during the exercise sessions as described below. The experimental sessions involved assessment of upper limb performance (Box and Block Test, pinch and grip strength) and TMS measures immediately before and after each exercise session. Oxygen uptake, carbon dioxide production, breathing frequency, and tidal volume were recorded breath-by-breath throughout the maximal graded exercise test and experimental sessions using an indirect calorimetry system (Moxus, AEI Technologies, Pittsburgh, PA). Heart rate was collected in line with respirometry data using a chest strap sensor (H10, Polar Electro Inc., NY, USA).

### Participants

Twelve chronic stroke survivors (>6 months) from an outpatient rehabilitation center were recruited. Participants had confirmed ischemic or hemorrhagic stroke, were able to ambulate with/without aid >10 m, received medical clearance for aerobic exercise, were able to follow two-step commands, and were eligible for TMS assessment based on a standardized TMS screening form (Rossi et al., 2009). Demographics, including age (years), gender, time since stroke (months), type of stroke (e.g., hemorrhagic or ischemic), brain lesion location (e.g., temporal lobe), and presence of co-occurring comorbid conditions (e.g., diabetes, hypertension) were collected. Stroke severity was determined by a physician using the National Institute of Health Stroke Scale (NIHSS; 0 = no symptoms; 1–4 = minor; 5–15 = moderate; 16–20 = moderate-severe; 21–42 = severe) (National Institute of Neurological, 2011), and hand spasticity level was determined by a physiotherapist using the Modified Ashworth Scale (Bohannon and Smith, 1987).

### Exercise protocols

#### VO_2max_ determination

Prior to completing the experimental sessions, participants performed a maximal graded exercise test on the total body recumbent stepper (NuStep, Ann Arbor, Michigan) to determine VO_2max_. Participants were instructed to not engage in structured physical activities for 24-h prior to each session, and to avoid consuming food or caffeine at least 2 h prior coming to the lab. The graded exercise test was adapted from previous work in this population (Billinger et al., 2008). Briefly, after familiarizing participants with the NuStep and adjusting the ergometer for arm and leg length, participants maintained 80 steps per minute while the load level was gradually increased to level 3. This workload (~20 Watts) was maintained for the first 2 min, which was then increased by one load level every 2 min at 80 strides per minute (~20 Watts increments) until exhaustion. If exhaustion was not reached after load level 10 (maximal load), the strides per minute was increased in increments of 10 every 2 min until exhaustion. The test was terminated using predefined criteria (Billinger et al., 2008; Ferguson, 2014): (i) volitional exhaustion, (ii) no increase in oxygen intake or heart rate despite increases in workload, (iii) inability to maintain workload, (iv) signs of excessive fatigue. Achievement of VO_2max_ was assessed based on attainment of at least two of the following criteria: (i) attainment of volitional exhaustion; (ii) a plateau in oxygen intake (< 80 ml min^−1^) despite increasing workload; (iii) respiratory exchange ratio (volume of dioxide oxygen expire/volume of oxygen intake) above 1.10; and (iv) maximal heart rate ± 10 beats min^−i^ of predicted maximum heart rate calculated as: 206.9−(0.67 × *age*)*or* 164−(0.7 × *age*) if prescribed beta blockers (Billinger et al., 2008; Ferguson, 2014).

#### Exercise intervention protocols

**Figure 1:**
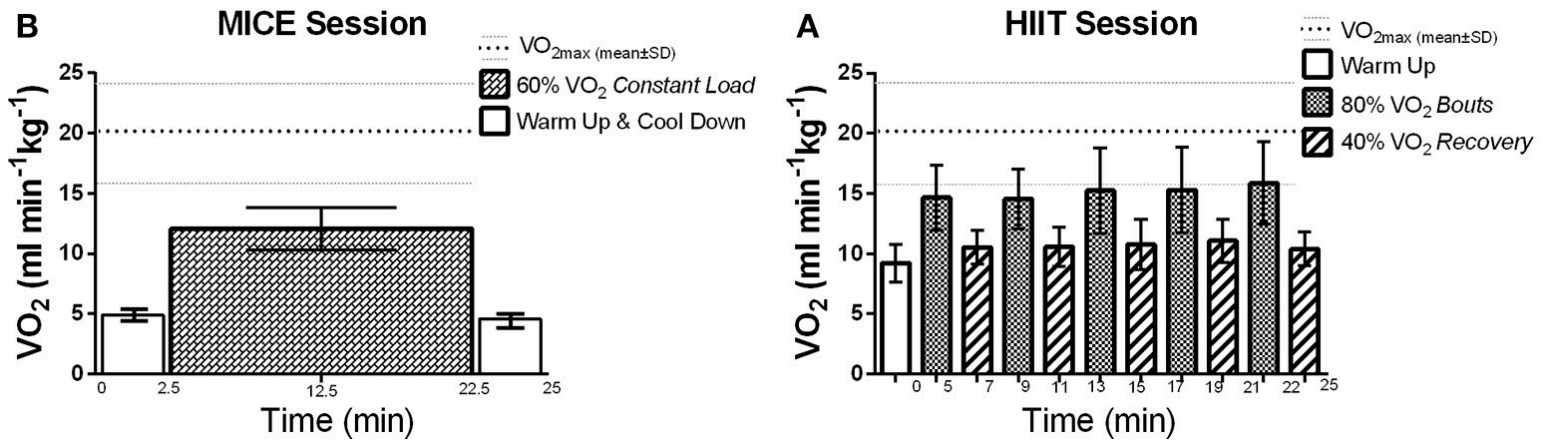
Average VO_2_ (ml min^−1^kg^−1^) reached during exercise sessions: VO_2max_: 20.44 ± 4.8 ml min^−1^kg^−1^, mean is represented by the thick dotted line and standard deviation (SD) is represented by the thinner dotted line. **(A)** HIIT session: At 40% VO_2_: 10.68 ± 1.6 ml min^−1^kg^−1^ (52.24 ± 1.3% of VO_2max_), and; at 80% VO_2_: 15.12 ± 2.9 ml min^−1^kg^−1^ (74 ± 2.59% of VO_2max_). **(B)** MICE session: at 60% VO_2_: 12.06 ± 1.8 ml min^−1^kg^−1^ (59.02 ± 1.8% of VO_2max_).

Each exercise intervention lasted 25 min on the NuStep, which included both the warm-up and cool-down phases. During these sessions, participants were instructed to only use their legs to maintain the workloads and the hand attachments were positioned out of reach. This protocol was employed to control for fatigue in the upper limb which could limit interpretation of TMS variables. For MICE, participants maintained a constant step cadence (60–80 strides per minute) at the load level that corresponded to 60% of their VO_2max_ for 20 min (Billinger et al., 2012; Ferguson, 2014) (Figure 1B). For HIIT, the workload was gradually increased during the warm-up phase until the load level corresponding to 80% of VO_2max_ was reached. After which, step cadence was maintained (60–80 strides per minute) while the load level was alternated every 2 min to achieve 80 and 40% VO_2max_ for the high-intensity and active recovery intervals, respectively (Figure 1A). Participants performed five cycles starting with the high intensity interval for a total of 20 min. The final active recovery interval also served as the cool-down phase for the HIIT intervention.

### Neurophysiological variables

#### Electromyography

Preceding and directly following exercise sessions, motor evoked potentials (MEP) were investigated from the resting first dorsal interosseous muscle in both hands. Foam surface electrodes (Kendall 200 Coviden, Mansfield, MA) were used to measure the electromyography activity from the first dorsal interosseous, using a bipolar configuration (Ag-AgCl, 2-cm inter-electrode distance). The ground electrode was placed on the medial epicondyle and the reference electrode was placed on the interphalangeal joint of the index finger. Participants had their skin shaved, abraded, and wiped with alcohol swabs to remove any hair, dead skin, and oil secretion, ensuring optimal quality of the electromyography signal. Signals were sampled at 40,000 Hz using a CED 1401 power interface (Cambridge Electronic Design 1401, Cambridge, UK) and amplified with a gain of 1000x and filtered with a 3-pole Butterworth filter with cut-off frequencies of 10–1,000 Hz (Cambridge Electronic Design 1902, Cambridge, UK). Data were recorded from 100 ms before to 200 ms after the TMS pulse.

#### TMS

Monophasic magnetic posterior-anterior pulses were delivered over the primary motor cortex area using a BiStim 200^2^ stimulator (Magstim Co. Whitland, UK) connected to a Double 70 mm figure-of-eight coil (Magstim, Co.). A neuronavigation device (Brainsight, Rogue Research Inc, Montreal, QC, Canada) was paired with the TMS to help guide the angle and direction of the coil over participants' scalp. The coil was maintained tangentially to the scalp with the handle pointing backwards and laterally at an angle of 45° from the midline perpendicular to the central sulcus. The Montreal Neurological Institute brain template was rendered into the BrainSight software and used as a 3-D stereotaxic template (Collins et al., 1994; Fonov et al., 2011). During the TMS sessions, participants were comfortably seated and instructed to remain alert and as relaxed as possible. First, the most responsive brain area responsible for the first dorsal interosseous muscle was found by firing the TMS at different locations over the participants' scalp. Each site was stimulated three times and the site with the highest averaged MEP amplitude was taken as the hotspot. The hotspot for each participant was recorded into the BrainSight software so that the same area is being stimulated in the subsequent TMS sessions. The resting motor threshold was determined as the minimum intensity required of the stimulator to elicit an MEP in the resting first dorsal interosseous muscle with a peak-to-peak amplitude of 50 μV or more in at least 5 out of 10 stimulations. Resting motor thresholds values were expressed as the maximal stimulator output percentage (MSO%), which ranges from 0 to 100 of MSO%.

#### TMS paired-pulse

To investigate intrinsic brain mechanisms of inhibition and facilitation, two TMS pulses, a conditioning stimulus and a test stimulus, separated by an interstimulus interval were delivered over the hotstpot. Short intervals (e.g., 2–5 ms) have been used to investigate short intracortical inhibition mechanisms, whereas longer intervals (e.g., 10–25 ms) have been used to investigate mechanisms of intracortical facilitation (Goss et al., 2012). The paired-pulse intervals used for this study were 3.0 and 12 ms, and the intensities used for the conditioned and the test stimulus were 80% of the resting motor threshold and the MSO% required to elicit an MEP with a peak-to-peak amplitude of 1 mV, respectively. There was a total of 60 stimulations: 10 trials of paired-pulses at each interval (total of 40 stimulations), and 20 single pulses recording the test stimulus alone. Stimulations were randomized.

#### Upper limb measures

Before and directly following the exercise and TMS experiment, upper limb dexterity was assessed using the box and block test (Mathiowetz et al., 1985), which requires participants to transport as many wooden blocks (dimensions: 2.5 × 2.5 × 2.5 cm) as possible, one at a time, from one box (dimensions: 53.7 × 25.4 × 8.5 cm) to another and crossing a 25.4 cm tall partition (i.e., obstacle) between boxes. The score is calculated as the number of transported boxes in a 1-min period, whereby higher scores indicate better manual dexterity. Grip and pinch strength were measured using a grip (Lafayette Instruments, Lafayette, IN) and a pinch dynamometer (B&L engineering, Santa Ana, CA). Participants were asked to perform grip and pinch maximal voluntary contractions and the strength was recorded in kilograms (Kg). Each upper limb test [strength (pinch and grip) and dexterity] was completed twice, alternating hands, and the scores were averaged.

### Data reduction and calculations

#### VO_2max_

As the metabolic cart system was set to record breath-by-breath data, a moving average of 10 data points was used to smooth and remove any noise from the VO_2_ and VCO_2_ data. In addition, visual inspection of the smoothed data for each exercise session for every participant ensured exclusion of data outliers. The absolute VO_2max_ was identified as the highest value for volume of oxygen uptake during the maximal graded exercise test from the smoothed data.

#### Exercise intensities and matching workloads

By using the metabolic values recorded in the maximal graded exercise test, the proposed intensities for HIIT (40%, and 80% of VO_2max_) and MICE (60% of VO_2max_) were calculated by using the formula = [(*VO*_2_max− *Resting VO*_2_) × (*intensity*_40, 60, *or* 80%_+ *Resting VO*_2_)] (Ferguson, 2014). To ensure that participants expended the same amount of energy (Kcal/session) by the end of both sessions, we matched the interventions for total workload performed and time exercising (i.e., 60% for 20 min = (40*% for* 10 min + 80*% for* 10 min).

#### Energy expenditure of exercise

The following equation was used: = (4.471 × *VO*_2_) − (0.55 × *VCO*_2_) (Jeukendrup and Wallis, 2005) to determine energy expenditure of exercise. For MICE, the steady-state values of VO_2_ and VCO_2_ were averaged. For HIIT, each of the 2-min bouts were inspected and the first 20–30 s following the change in intensity were removed to avoid calculation of major drifts between the changes in intensities, and VO_2_ and VCO_2_ were calculated from the remaining data. The same procedure was performed for the warm up and cool down phases for both exercise sessions. The total energy expenditure during the sessions (kcal/session) was found by multiplying the energy expenditure in kcal/min of each of the exercise phases by its total time and adding them together to ensure that the entire 25 min of exercise was considered.

#### MEPs

MEPs were visually inspected such that signals with high electromyography background activity (>20% of the mean) preceding the TMS stimulus were excluded from analysis. MEP amplitude was calculated as peak-to-peak from the averaged valid MEPs (Rossini et al., 2015). MEP latency, which is a measure of nerve conduction speed, was calculated as the time in milliseconds from TMS artifact (0 ms) to the MEP onset (McCambridge et al., 2015) using 10 averaged test stimuli. For paired-pulse experiments, the magnitude of the inhibition or facilitation was reported as a percentage of the unconditioned MEP (i.e., test stimulus), where values < 100% represent brain inhibition, whereas values >100% represent brain facilitation (Goss et al., 2012; Nepveu et al., 2017). Data were collected and analyzed with Signal 6.0 software (Cambridge Electronic Design, Cambridge, UK).

### Statistical analysis

Prior to testing for differences in the primary outcome measures, we first evaluated whether HIIT and MICE, (1) achieved the predetermined intensity levels, and (2) were matched for total energy expenditure. A paired-*t*-test was performed to analyze baseline differences in TMS and upper limb measures between sides, as well as the total energy expenditure performed in both sessions.

A two-way repeated measures ANOVA with Time (Pre and Post) as the first within subject factor and Session (MICE and HIIT) as the second within subject factor was used to test for exercise induced effects on CSE measures (resting motor threshold, paired-pulses at 3 ms, and at 12 ms, and MEP Latency) and the upper limb measures (Box and Block Test, pinch and grip strength). These RM-ANOVAs were performed separately for ipsilesional and contralesional hemispheres and the more-affected and less affected hands. Significance was set at an alpha level of *p* < 0.05 for all statistical tests. For significant interactions between Time × Session, pairwise analyses were performed. Paired *t*-tests or non-parametric paired *t*-tests were run depending on normality of the variable. To control for family-wise error, Bonferroni corrections were used for multiple comparisons.

Spearman's correlations were used to test for relationships between exercise-induced changes in CSE and upper limb function. Lastly, associations between energy expenditure of exercise (the control variable) and changes in CSE and upper limb function were investigated. Statistical analysis was performed on SPSS 23.0 (IBM Corporation, Armonk, New York). Graphs were created with Graph-Pad (Prism, version 6). Data are reported as mean ± SD.

## Results

Ten males and two females (age, 62.5 ± 9.0 years; range: 46–77) with chronic stroke (months since stroke: 26.67 ± 22.0; range: 6–72) participated in the current study. Participants had residual impairments (NIHSS = 3.33 ± 3.0, range: 1–10), all were right-handed, and 50% had their dominant side affected by stroke. One participant had hemorrhagic and 11 had ischemic stroke. As displayed in Table 1, hand spasticity scores (Modified Ashworth scale) showed that all but three subjects had spasticity of the hand.

**Table 1:** Participants characteristics.

**Subject number**	**Gender**	**Handedness**	**Affected hemisphere**	**Type and brain location of stroke**	**Months since stroke**	**Age (years)**	**Stroke severity (NIHSS)**	**Hand spasticity (MAS)**
1	M	Right	Left	Ischemic. temporal/parietal lobe	27	55	7	0
2	M	Right	Left	Ischemic. posterior internal capsule	8	72	1	0
3	F	Right	Right	Ischemic. thalamus	8	67	1	1
4	F	Right	Left	Ischemic. parietal lobe	51	62	1	3^*^
5	M	Right	Right	Ischemic. frontal/temporal lobe	15	46	3	3^*^
6	M	Right	Right	Ischemic. medulla	13	63	10	0
7	M	Right	Right	Hemorrhagic. basal ganglia	59	77	7	1+
8	M	Right	Right	Ischemic. frontal lobe (right)/corona radiata (left)	26	53	2	1+
9	M	Right	Right	Ischemic. corona radiata	6	67	1	1+
10	M	Right	Left	Ischemic. basal ganglia/intern. capsule/corona radiata	72	65	3	3^*^
11	M	Right	Left	Ischemic. basal ganglia/parietal lobe	17	69	1	3
12	M	Right	Left	Ischemic. insula/temporal/parietal lobes	18	54	3	3^*^
				Mean±SD:	26.67 ± 22.0	62.5 ± 9.0	3.33 ± 3.0	1.78 ± 1.5

MAS, Modified Ashworth Scale; NIHSS, National Institutes of Health Stroke Scale.

* *participants with no MEPs recorded in the affected side*.

All participants were able to perform the maximal graded exercise test protocol until volitional exhaustion. As reported in Table 2, all but one participant reached the criteria indicating that maximal exertion was achieved. VO_2max_ was 20.44 ± 4.8 ml min^−1^ kg^−1^ for this cohort with most participants scoring at the very lowest percentile of fitness compared to their age and gender matched peers (Ferguson, 2014). During the MICE intervention, participants maintained workloads that corresponded to 59 ± 2% of their VO_2max_, which was very close to the target intensity of 60%. As expected, more variability was recorded between target and observed values during HIIT. The observed intensity for the high-intensity and recovery intervals were 74 ± 3 and 52 ± 1%, respectively (Figure 1). However, as reported in Table 3, the total amount of work performed was closely matched between the two interventions, with no significant differences between the total energy expended on both exercise sessions. All participants were able to perform both HIIT and MICE sessions without adverse events.

**Table 2:** Fitness profile of participants.

**Subject**	**BMI**	**HR_max_ (beats/min)**	**VO_2max_ (ml.min^−1^kg^−1^)**	**Aerobic Fitness (% of Normative Data)**	**% of Predicted HR_max_**	**RER at VO_2max_ (VCO_2_/VO_2_)**
1	32.5	125	18.81	<1	100^†^	1.07
2	32.6	122	15.77	<1	77	1.16
3	25.1	120	14.26	<1	103^†^	1.03
4	26.3	143	14.31^*^	<1	87	1.07
5	22.8	185	24.94	<1	105	1.07
6	35.9	162	25.16	15	98	0.98
7	28.4	137	17.94	1	124^†^	0.99
8	32.0	168	24.35	5	98	1.01
9	29.8	154	20.19	1	95	1.06
10	27.6	176	29.48	30	108	1.1
11	27.9	173	21.21	5	108	1.06
12	29.1	157	18.88	<1	92	1.2
Mean±SD:	29.2 ± 3.6	152 ± 22	20.44 ± 4.8	–	100 ± 12	1.07 ± 0.1

RER, Respiratory Exchange Ratio; BMI, Body mass Index; HRmax, maximal heart rate; VO2max, maximal volume of oxygen intake. Aerobic Fitness is ranked as % of normative data according to American College Sports of Medicine (Ferguson, 2014).

† Participants taking beta blockers.

* *Participant did not meet the criteria for achieving VO2max based on RER 1.1 and maximal heart rate ± 10 beats min^−1^ of predicted maximum heart rate calculated as: 206.9–(0.67 × age) (Ferguson, 2014)*.

**Table 3:** MICE and HIIT metabolic data.

**Energy expenditure (Kcal/min)**		
**Exercise phase**	**MICE**	**HIIT**
Warm up	3.49 ± 0.63	3.39 ± 0.55
40% VO_2max_	–	3.82 ± 0.72
60% VO_2max_	4.58 ± 1.08	–
80% VO_2max_	–	5.49 ± 1.46
Cool down	3.53 ± 0.89	–
Total EEE (Kcal/session):	109.28 ± 24.5	109.72 ± 23.8

*MICE, moderate intensity constant-load exercise; HIIT, high intensity interval training; VO_2max_, maximal volume of oxygen intake; EEE, energy expenditure of exercise*.

**Figure 2:**
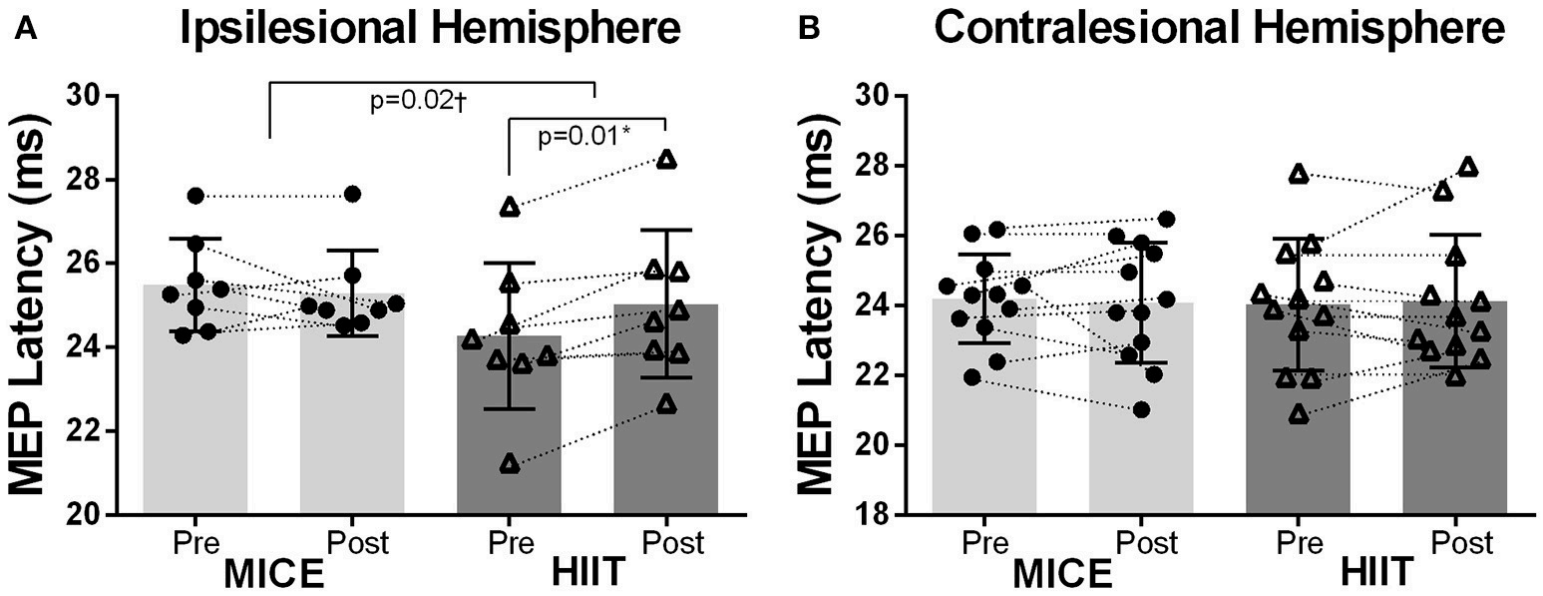
Changes in MEP latency following exercise. **(A)** There was a significant difference in MEP latency changes between MICE and HIIT in the ipsilesional hemisphere. MEPs were conducted slower after HIIT but not after MICE. **(B)** There were no changes in the contralesional hemisphere. *Significant pre and post MEP latency changes after HIIT. ^†^Significant differences in MEP latency changes (Δ = *post* − *pre*) between MICE and HIIT.

MEPs were recorded in the less affected hand through stimulation of the contralesional hemisphere in all subjects. However, MEPs were not observed in the affected hand after stimulation of the ipsilesional hemisphere in 4 participants (subjects 4, 5, 10, and 12) before or after the interventions. Also, these four subjects were unable to perform the upper limb tests using the affected hand due to severity of hand spasticity. The resting motor thresholds were similar between the ipsilesional and contralesional hemispheres prior to exercise in both treatments. No group or time effects were observed for resting motor thresholds after exercise sessions in the ipsilesional [*F*_(1, 7)_ < 3.73, *p* > 0.10] or contralesional hemispheres [*F*_(1, 11)_ < 0.24, *p* > 0.63). A significant Group (MICE, HIIT) × Time (pre, post) interaction was observed for MEP latency in the ipsilesional [*F*_(1, 7)_ = 8.56, *p* = 0.02] but not the contralesional hemisphere [*F*_(1, 11)_ = 0.22, *p* = 0.65]. *Post-hoc* analysis revealed a significant lengthening of MEP latency after HIIT (Figures 2A,B) while the other comparisons did not differ.

**Figure 3:**
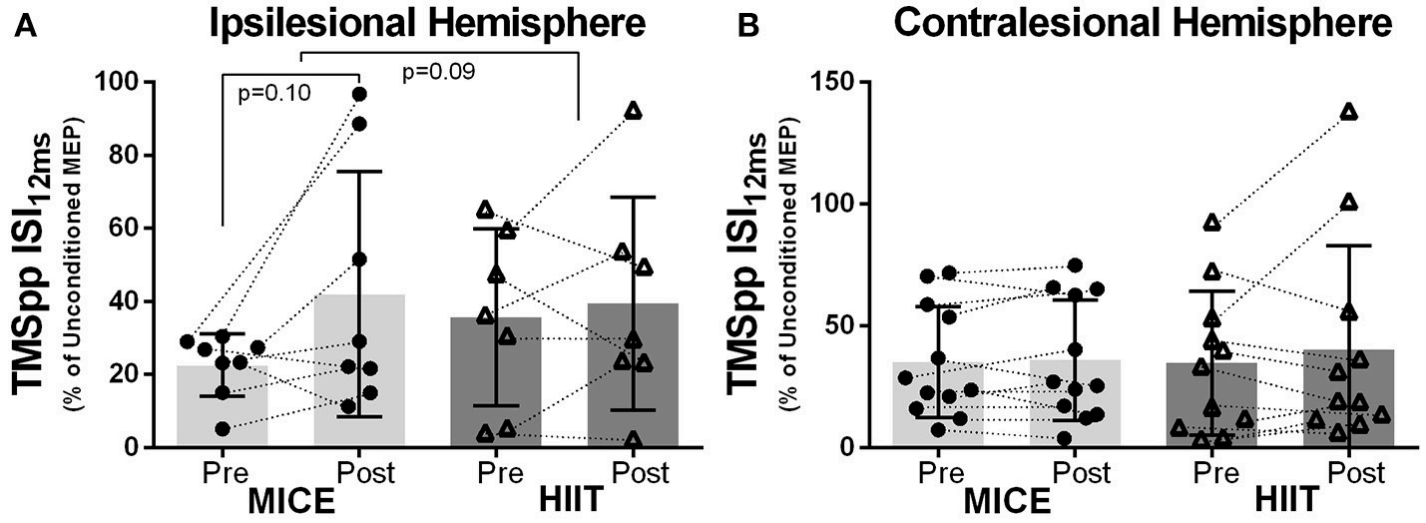
Exercise-induced changes in MEP amplitude: **(A)** ipsilesional and **(B)** contralesional hemispheres after MICE and HIIT using TMS paired-pulse with an interstimulus interval of 12 ms (ISI_12ms_) between pulses.

No significant effect of MICE or HIIT was observed in the ipsilesional or contralesional hemispheres for intracortical mechanisms assessed with TMS paired-pulses. However, both exercise interventions led to small decreases in brain inhibition, although not significant, using 12 ms interstimulus interval (Figures 3A,B). As seen in Table 4, the TMS paired-pulse protocol using the 12 ms interstimulus interval did not elicit intracortical facilitation in this cohort.

**Table 4:** Transcranial magnetic stimulation (TMS) values and upper limb scores.

**Exercise session:**			**MICE**						**HIIT**					
**Brain hemisphere:**			**Ipsilesional (*****n*** = **8)**			**Contralesional (*****n*** = **12)**			**Ipsilesional (*****n*** = **8)**			**Contralesional (*****n*** = **12)**		
**Pre, post, and change:**			**Pre**	**Post**	**Change**	**Pre**	**Post**	**Change**	**Pre**	**Post**	**Change**	**Pre**	**Post**	**Change**
TMS Measures	Single pulses	RMT (MSO%)	44.62 ± 8.1	42.0 ± 8.5	−2.62 ± 7.3	44.17 ± 8.4	43.17 ± 6.7	−1.08 ± 4.6	39.75 ± 7.9	39.12 ± 6.3	−0.75 ± 5.0	43.83 ± 7.6	44.75 ± 11.1	0.58 ± 6.0
		MEP Latency (ms)	25.49 ± 1.1	25.29 ± 1.0	−0.21 ± 0.7^†^	24.18 ± 1.3	24.08 ± 1.72	−0.10 ± 1.0	24.27 ± 1.8	25.04 ± 1.8^*^	0.76 ± 0.6^†^	24.02 ± 1.9	24.11 ± 1.9	0.09 ± 1.0
	TMSpp (%Unconditioned MEP)	ISI_3ms_	13.78 ± 9.3	26.78 ± 23.1	11.56 ± 15.2	21.50 ± 13.0	23.48 ± 17.00	1.79 ± 9.7	22.61 ± 17.3	30.32 ± 32.1	7.71 ± 26.2	28.21 ± 25.8	29.98 ± 27.5	1.78 ± 34.6
		ISI_12ms_	22.48 ± 8.5	41.98 ± 33.6	19.50 ± 28.9	35.10 ± 22.8	35.90 ± 24.83	0.81 ± 7.8	35.65 ± 24.3	39.41 ± 29.2	3.76 ± 20.3	34.67 ± 29.5	40.35 ± 42.6	5.68 ± 21.9
**Hand:**			**Affected (*****n*** = **8)**			**Less affected (*****n*** = **12)**			**Affected (*****n*** = **8)**			**Less affected (*****n*** = **12)**		
**Pre, post, and change:**			**Pre**	**Post**	**Change**	**Pre**	**Post**	**Change**	**Pre**	**Post**	**Change**	**Pre**	**Post**	**Change**
Upper limb performance	Strength	Pinch (Kg)	8.96 ± 1.9	8.41 ± 2.0^*^	−0.55 ± 0.5	8.88 ± 2.3	8.70 ± 2.22	−0.18 ± 0.6	8.83 ± 2.0	8.65 ± 2.2	−0.18 ± 0.5	8.49 ± 2.5	8.35 ± 2.4	−0.14 ± 0.6
		Grip (Kg)	26.19 ± 6.4	26.62 ± 7.0	0.43 ± 3.7	30.79 ± 9.1	30.29 ± 8.03	−0.50 ± 3.0	27.56 ± 7.4	27.31 ± 7.0	−0.025 ± 1.0	30.96 ± 9.4	30.83 ± 8.6	−0.08 ± 2.0
	Dexterity	BBT (seconds)	51.12 ± 11.3	52.19 ± 11.0	1.06 ± 3.9	57.46 ± 10.2	59.33 ± 9.5	1.92 ± 5.5	50.25 ± 10.9	50.44 ± 9.8	0.19 ± 5.8	58.04 ± 7.8	58.29 ± 8.9	0.25 ± 4.8

MEP, Motor Evoked Potential; RMT, Resting Motor Threshold eliciting a 0.05mV MEP; TMSpp, TMS paired-pulse; ISI12ms, Interstimulus Interval of 12ms; ISI3ms, Interstimulus Interval of 3.0ms, averaged; BBT: box block test; Change (Δ = post – pre).

* Significant different from pre (Bonferroni corrected p < 0.01);

† Change is significantly different between MICE and HIIT (p < 0.05).

*Ipsilesional and affected hand's data could not be collected in 4 participants due to severity of hand spasticity (n = 8); Contralesional and less affected hand's data could be collected in all participants (n = 12)*.

Although there was no main effect of Session [*F*_(1, 7)_ = 0.02, *p* = 0.88] or Session × Time interaction [*F*_(1, 7)_ = 3.39, *p* = 0.11], a significant decrease in pinch strength was observed after MICE in the affected hand [*F*_(1, 7)_ = 6.56, *p* = 0.04]. For the remaining upper limb measures (grip strength and Box and Block Test), we did not observe main effects of Time, Session or Time × Session interactions.

### Relationships between CSE, upper limb function, and total energy expenditure of exercise

**Figure 4:**
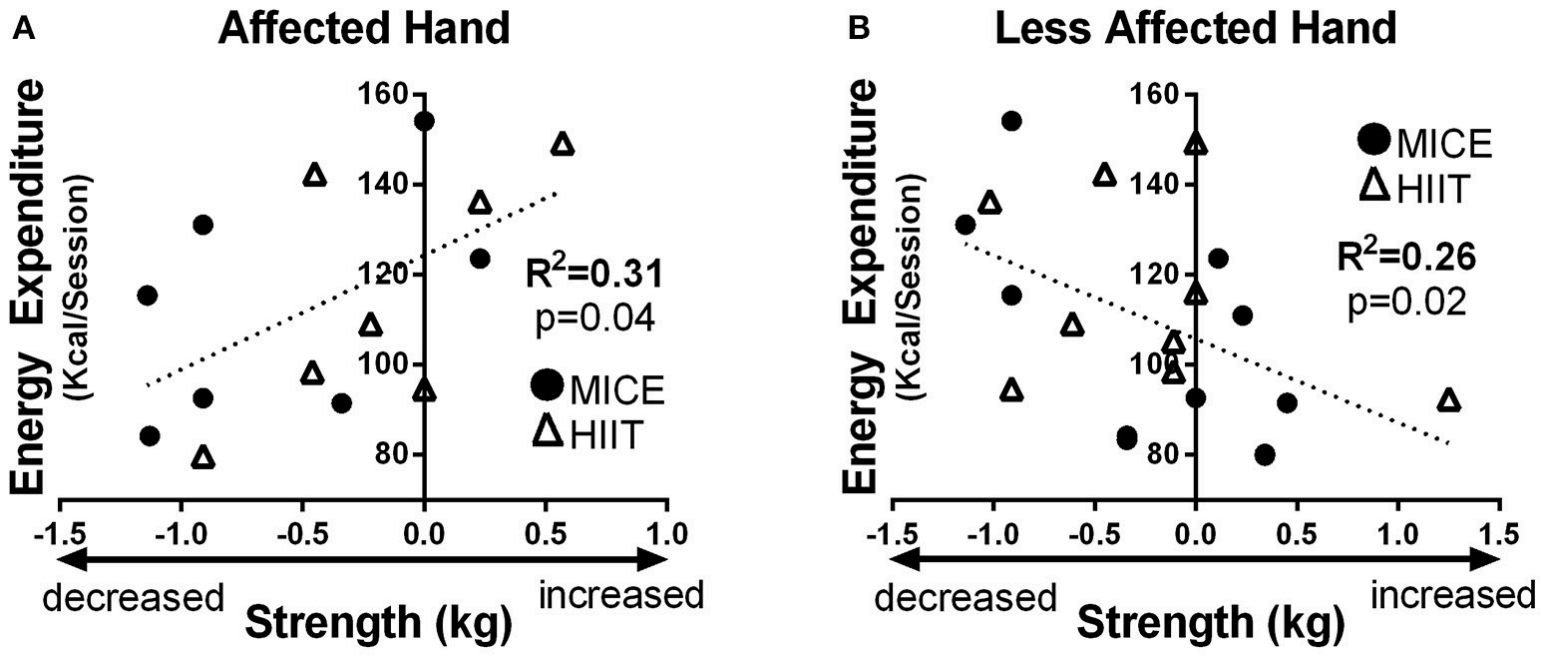
Total energy expenditure of exercise related to: **(A)** enhanced pinch strength in the affected hand, and; **(B)** decreased pinch strength in the less affected hand. Solid circles MICE (moderate intensity continuous exercise), open triangles HIIT (high intensity interval training).

No intensity-dependent associations were observed between CSE changes and upper limb function. However, higher total energy expenditure of exercise was related to increased pinch strength in the affected hand after exercise when combining HIIT and MICE sessions (*R*^2^ = 0.31, *p* = 0.04) (Figure 4A). In the less affected hand, the opposite effect was observed, higher total energy expenditure correlated with decrease in pinch strength (*R*^2^ = 0.26, *p* = 0.02) (Figure 4B).

## Discussion

The primary objective of the current study was to investigate the effects of single bouts of MICE and HIIT on CSE in chronic stroke survivors. In an attempt to evaluate intensity dependent effects, the exercise treatments were matched for both duration of activity and total energy expenditure. To the best of our knowledge, this is among the first studies to evaluate acute changes in CSE using this experimental approach in stroke survivors. When exercise duration and total energy expenditure were matched, we observed no differences between exercise sessions in typical TMS CSE metrics; resting motor threshold and intracortical mechanisms evaluated with TMS paired-pulses. However, intensity dependent effects were observed in MEP latency with a longer nerve conduction time between the TMS stimulation to the MEP onset elicited from the ipsilesional hemisphere and measured in the contralateral affected hand. Although increases in MEP amplitudes were observed for some participants using the TMS paired-pulse at 12 ms after MICE, the overall time effect was not significant. The contralesional hemisphere was relatively resistant to exercise-induced effects. Although a decrease in pinch strength was observed in the affected hand after MICE, this change was not different from the HIIT intervention and no other changes in upper limb measures were detected. Interestingly, when collapsing both exercise sessions, greater total energy expenditure was associated with enhanced pinch strength after exercise in the affected hand and the inverse association was observed in the less affected hand. Acute changes in CSE following the exercise sessions did not correlate with changes in measures of upper limb strength or dexterity.

### No effects of acute aerobic exercise on resting motor threshold

In general, CSE represents the summation of both inhibitory and excitatory inputs on descending neurons (Ziemann et al., 2015). Resting motor threshold is used to quantify levels of CSE *in vivo*; with higher MSO% needed to elicit a MEP in individuals with lower levels of CSE and representing a measure of motor neurons membrane excitability (Boroojerdi et al., 2001; Ziemann et al., 2015). We found no effect of our exercise interventions on resting motor threshold. This aligns with previous work in healthy individuals (Singh et al., 2014; Neva et al., 2017). Similar findings were also reported in stroke survivors after performing maximal aerobic exercise (Nepveu et al., 2017). In contrast, a decrease in CSE of the ipsilesional hemisphere and an increase in the CSE of the contralesional hemisphere was reported in chronic stroke survivors after HIIT (Madhavan et al., 2016). However, these changes were evaluated using suprathreshold single pulse stimulations (i.e., >100% of the resting motor threshold) used to assess the function of higher-threshold motor neurons deeper in the corticospinal tract that are controlled by glutamatergic and noradrenergic neurotransmission (Boroojerdi et al., 2001; Ziemann et al., 2015). These mechanisms can be easily modulated by an acute bout of exercise (Basso and Suzuki, 2017). Nonetheless, using suprathreshold stimuli, previous research in chronic stroke has shown that low intensity continuous aerobic exercise did not elicit CSE changes (Murdoch et al., 2016). These findings suggest that an acute bout of high intensity may be superior to low intensity aerobic exercise to modulate CSE by increasing glutamate and noradrenergic neurotransmission in chronic stroke. However, as shown here and in agreement with other studies in healthy and stroke population, resting motor threshold as measure of membrane excitability that is modulated by voltage gated Na^+^ channels (Boroojerdi et al., 2001), may be less likely to change acutely after a bout of aerobic exercise. Conversely, long-term exercise training increases CSE (Goodall et al., 2014; Kidgell et al., 2017), and trained individuals have lower motor thresholds when compared to untrained (Monda et al., 2017). This suggests that mechanisms behind resting motor thresholds may require further exercise training long-term adaptations.

### Nerve conduction latency

In stroke survivors, shortened MEP latency is associated with better recovery of both upper (Turton et al., 1996; Platz et al., 2005; Barker et al., 2012) and lower extremities (Beaulieu et al., 2014). Physical rehabilitation interventions aiming to reduce MEP latency have primarily focused on training induced effects (e.g., after 12 sessions over 4 weeks) using repetitive task-oriented exercise (Beaulieu and Milot, 2017). However, clinical trials delivering task-specific training alone were not able to demonstrate shortening of MEP latency in chronic stroke (Stinear et al., 2008). Conversely, pairing task-specific training with muscle stimulation techniques might be sufficient to reduce MEP latency and improve hand function in chronic stroke (Tarkka and Kononen, 2009; Barker et al., 2012). In either case, limited data is currently available describing the acute and long-term effects of aerobic exercise interventions on MEP latency in stroke survivors.

The current analysis revealed a lengthening of MEP latency after a single bout of HIIT while no significant changes were observed after MICE. Although we hypothesized an intensity dependent effect, the direction of the change was opposite to that anticipated and was only observed in the ipsilesional hemisphere. However, exercise-induced changes in MEP latency may be influenced by recent activity levels. A study by Forrester et al. (2006) also reported a lengthening of MEP latency after a single bout of treadmill exercise but the effect was only observed in trained stroke survivors (Forrester et al., 2006). Indeed, participants in the current study were more active than the general stroke population as indicated by their relatively higher VO_2max_ scores (Nepveu et al., 2017). Clarifying the relationships between fitness, intensity and exercise-induced changes in MEP latency is worthy of future investigation.

Lengthened nerve conduction time after HIIT could be explained by the cross-over effect of exercise on CSE. We examined CSE in a non-exercised muscle. In fact, CSE changes were recently observed in the non-exercised upper limb of healthy individuals after fatiguing exercise performed in the lower limb (Aboodarda et al., 2017). Although we did not measure exercise-induced fatigue, there is a pathophysiological adaptation that occurs after stroke leading to an increased proportion of highly fatigable (type II-like or fast-twitch) muscle fibers in the affected limbs (De Deyne et al., 2004; Severinsen et al., 2016). This morphological change is associated with a decreased muscle fiber cross-sectional area and a loss of non-fatigable (type I-like or slow-twitch) muscle fibers (Chokroverty et al., 1976; Landin et al., 1977; Jakobsson et al., 1991; Hachisuka et al., 1997). Also, during HIIT greater levels of muscle contraction in the legs are required to perform the high intensity bouts involving predominant recruitment of fatigable muscle fibers (Russell et al., 2003; Altenburg et al., 2007; Egan et al., 2010; Kohn et al., 2011). Accordingly, MEP latency lengthening was observed in the affected limb and only after HIIT. Alternatively, an exercise induced reduction in spasticity may also explain the current findings. previous research in stroke survivors demonstrated that 10 min of arm-cycling at 50% of maximal workload using the less-affected arm reduced spasticity of the non-exercised hemiplegic arm (Sakamoto et al., 2014). Since spasticity is characterized by an increase in muscle tone, and MEPs are conducted faster during muscle contraction (Hess et al., 1987), a plausible explanation for the lengthening in MEP latency was reduced spasticity after HIIT. There is limited evidence, however, demonstrating relationships between spasticity and MEP latency in chronic stroke (Cakar et al., 2016), and we did not measure exercise-induced changes in spasticity. In either case, more studies are warranted to fully understand the underlying mechanisms behind the intensity-dependent changes of exercise on CSE and their long-term effects on functional recovery in chronic stroke survivors.

### No effects of exercise on brain inhibition and facilitation

GABA is the main inhibitory neurotransmitter in the brain, and decreasing its activity is crucial for inducing long-term potentiation (Kujirai et al., 1993; Ziemann et al., 1996a). Also, the downregulation of GABA has been associated with an increase in facilitatory mechanisms that are mediated by the upregulation of glutamate, the main excitatory neurotransmitter (Goss et al., 2012), suggesting that inhibitory and facilitatory processes are not mutually exclusive. In stroke, the fine-tuning of GABA mediated inhibitory networks may play a role in the induction of neuroplasticity (Taubert et al., 2015) and functional reorganization of both hemispheres (Manganotti et al., 2008; Takechi et al., 2014). TMS studies in healthy participants have shown that acute aerobic exercise can downregulate brain inhibition (Molteni et al., 2002; Singh and Staines, 2015) and upregulate brain facilitation (Singh and Staines, 2015).

Despite the numerous TMS studies investigating GABAergic and glutamatergic intracortical mechanisms by using TMS paired-pulses paradigms (Goss et al., 2012; Vucic and Kiernan, 2017), it is still unclear which cortical mechanisms are being elicited during TMS paired-pulses in stroke (Liepert et al., 1998; Reis et al., 2008). The current study used similar TMS paired-pulse protocols to those used previously (Manganotti et al., 2002; Nepveu et al., 2017). However, we observed very high values of intracortical inhibition when using typical interstimulus interval of 3 ms, and a lack of intracortical facilitation when using an interstimulus interval of 12 ms. It is conceivable that the intensity used for the subthreshold stimulus (i.e., 80% of resting motor threshold) could have been too high, thus instead of acting on the surface inhibitory interneurons, could have depolarized the motor neuron, creating refractoriness during the subsequent suprathreshold stimulus. It might have been preferable to use an intensity below the active motor threshold as suggested by majority of authors (Liepert et al., 1998, 2000; Cicinelli et al., 2003; Swayne et al., 2008; Edwards et al., 2009; Murdoch et al., 2016; Neva et al., 2016). Also, the interstimulus intervals required to elicit intracortical facilitation and inhibition may vary between individuals regardless stroke (Du et al., 2014). So, it is possible that the unique structure brain reorganization that occurs after stroke may require patient-specific interstimulus intervals.

Although we were not able to assess intracortical mechanisms of facilitation using the interstimulus of 12 ms (all values < 100% of unconditioned MEP), increased MEP amplitudes were observed after MICE in the ipsilesional hemisphere in 6 out of 8 subjects using this protocol. As previously reported, paired suprathreshold levels can produce facilitatory effects in the spinal cord that can last up to 20 ms (Cowan et al., 1986). Therefore, although unintended, our subthreshold stimulus may have been sufficient to drive descending corticospinal volleys and we could have still detected facilitation. Previous research has shown that increased intracortical inhibition correlates with depressed intracortical facilitation (Ziemann et al., 1996b, 2015). In accordance, among healthy individuals moderate aerobic exercise decreased intracortical inhibition and increased intracortical facilitation (Singh et al., 2014; Smith et al., 2014). However, in chronic stroke, Nepveu and group (Nepveu et al., 2017) did not detect any effect on intracortical mechanisms of inhibition and facilitation after an acute bout of maximal aerobic exercise (i.e., maximal graded exercise test). Nonetheless, these authors showed that there was a change in the ratio between hemisphere's intracortical inhibition (contralesional/ipsilesional), whereby the ipsilesional hemisphere decreased in inhibition whereas the contralesional did not change. We could not find a difference in this same ratio. We noted a high degree of variability in response to the paired-pulse experiment in our participants, which may reflect their heterogeneity. In participants with severe hand spasticity (Modified Ashworth scale = 3 in 5 out of 12 participants) we could not quantify levels of inhibition or facilitation. However, ipsilesional/contralesional effects reported by Nepveu et al. partially agrees with our study, in that only the ipsilesional hemisphere was modulated after aerobic exercise.

There are several important differences between our study and the one described by Nepveu et al. (2017). First, these authors assessed CSE after maximal graded exercise test and our study assessed responses after MICE and HIIT. Secondly, our participants' residual impairment (NIHSS) and spasticity scores (Modified Ashworth scale) along with their Chedoke-McMaster Stroke Assessment score (mean 5.8) indicated that our participants were more disabled. Also, although the same TMS paired-pulse protocol was used in terms of muscle assessed (first dorsal interosseous), interstimulus interval and MSO% intensities, these authors used a different TMS stimulator, potentially delivering distinct pulses intensities (i.e., voltage), especially during paired-pulses (Sinclair et al., 2006; Goss et al., 2012). Finally, although the same recumbent bike was used (NuStep), our participants did not use their hands during the exercise, and this could have impacted CSE responses. Similarly, in chronic stroke, Murdoch et al. (2016) also investigated intracortical inhibition in the first dorsal interosseous muscle following continuous low-intensity cycling and could not show any intracortical inhibition changes.

Therefore, the intensity and type of exercise, the characteristics of the stroke population being assessed, and whether the target muscle participates in the exercise, are all factors that may play a role in detecting GABAergic and glutamatergic changes post-exercise. Taken together, these results support the hypothesis that aerobic exercise at moderate-high, but not low intensity levels may act as a tool to modulate the preserved long-term potentiation-like mechanisms in the ipsilesional hemisphere of chronic stroke survivors. Previous work in stroke suggest that increasing CSE of the ipsilesional hemisphere leads to improvement in both motor skill acquisition (Kim et al., 2006) and function (Hummel and Cohen, 2005) in chronic stroke, supporting the concept that aerobic exercise could be used to “prime” the brain when paired with other types of training.

### Intensity-dependent effects of exercise on upper limb function

The current analysis revealed no significant effect of MICE or HIIT on hand function. This contrasts with our previous findings in a similar stroke population where 20-min of body-weight supported treadmill exercise at 70% of heart rate reserve was sufficient to improve hand function. However, both studies found significant associations between changes in affected hand's function and the total energy expenditure of exercise. Interestingly, in the current study, bilateral measures allowed us to note an opposite relationship for the less-affected hand. This phenomenon may be explained by the interhemispheric competition model, which suggests that there is a relationship between hemispheres whereby decreasing function of one side is associated with improved performance on the contralateral side (Werhahn et al., 2002; Floel et al., 2008). In fact, reducing interhemispheric competition through brain stimulation methods by inhibiting the contralesional hemisphere and/or increasing CSE of the ipsilesional hemisphere, improves function of the affected hand (Nowak et al., 2009; Kakuda et al., 2011; Di Pino et al., 2014). These findings suggest that exercise induced changes in hand function may be related to the total energy expenditure of exercise rather than the relative exercise intensity itself. Also, given that opposing effects were observed in hand function, these exercise-induced changes are likely mediated through interhemispheric mechanisms. However, more studies are needed to confirm these results.

### Limitations

There were limitations in this study. In four participants, TMS variables could not be investigated due to the absence of MEPs among patients with more severely affected hands. Together with our limited sample size, loss of these data points may have compromised our ability to detect exercise effects. Also, although we used a protocol to elicit brain intracortical mechanisms of inhibition and facilitation, we could not observe intracortical facilitation in this cohort. We deliberately chose a heterogeneous group to better understand how aerobic exercise would impact CSE in a typical stroke population, however, this may have influenced our ability to detect statistically significant differences. Lastly, we only investigated acute exercise induced changes in CSE and their relationship with hand function and, therefore, these findings may not be applicable to current clinical stroke rehabilitation practice.

## Conclusion

The only exercise-induced change in CSE observed in the current study was lengthened nerve conduction latency. This effect was intensity-dependent as it was only observed after HIIT. CSE was not changed as measured by resting motor threshold and intracortical mechanisms of inhibition and facilitation assessed with TMS paired-pulses. In fact, the typical TMS paired-pulse protocol utilizing 12 ms-interval between pulses was not optimal for assessing intracortical facilitation in this cohort of chronic stroke survivors, rather, intracortical inhibition was noted. Although increases in MEP amplitude were observed after MICE for some participants using the 12 ms-interval, group changes did not reach the level of statistical significance. There were no intensity-dependent associations between CSE changes and upper limb function. However, regardless the type (HIIT or MICE) of exercise performed, we showed that higher energy expenditure of exercise correlated with enhanced strength in the affected hand and with decreased strength in the less affected hand. More studies are needed to fully understand the role of energy expenditure of exercise and the long-term effects of such exercise interventions, especially HIIT, in promoting neuroplasticity and functional recovery in stroke survivors.

## Author contributions

BA designed the experiment and collected the data. AC performed data entry, cleaned, and analyzed the data, interpreted the findings, wrote, edited, and submitted the manuscript. LK wrote and edited the manuscript. EW recruited participants and edited the manuscript. KW edited the manuscript. JM screened participants. MP conceived of and designed the experiment, screened subjects, and edited manuscript.

### Conflict of interest statement

The authors declare that the research was conducted in the absence of any commercial or financial relationships that could be construed as a potential conflict of interest.

## Abbreviations

CSE: corticospinal excitability
FDI: first dorsal interosseous
HIIT: high intensity interval training
MAS: Modified Ashworth Scale
MEP: motor evoked potential
MICE, moderate: constant-load exercise
MSO: maximal stimulator output
NIHSS: National Institute of Health Stroke Scale
TMS: transcranial magnetic stimulation
VCO_2_: volume of dioxide oxygen
VO_2_: volume of oxygen
VO_2max_: maximal volume of oxygen uptake.
